# Building a culture of research impact assessment within the agro-food research organizations

**DOI:** 10.1186/s40100-021-00204-5

**Published:** 2021-10-04

**Authors:** Bouali Guesmi, José M. Gil

**Affiliations:** Center for Agro-Food Economics and Development-UPC-IRTA (CREDA), Parc Mediterrani de la Tecnologia, Edifici EEABB-D4, 08860 Castelldefels, Barcelona Spain

The agro-food sector is an essential sector striving to satisfy the increasing demand for agro-food products worldwide. Accordingly, the Food Systems Summit has made a significant effort to heighten social and political awareness about the future food systems’ role in achieving the Sustainable Development Goals (SDGs) Agenda by 2030. Several research studies provide supporting evidence that research and development (R&D) investment is a key factor among others that contribute to enhance agricultural productivity growth and to generate high value of research return (Alston et al. 2010). However, a limited R&D budget, in both developed and developing countries has led to seriously underfunding of some emerging agro-food research areas. The recent COVID-19 crisis may have exacerbated this phenomenon.

In the past, the transformation processes of the agro-food system have received attention from policy-makers and stakeholders to achieve a more productive, sustainable, equitable and fairer system. Nowadays, growing social and political concerns about the effectiveness of public & private agro-food R&D make it necessary to evaluate the ability to deliver societal impacts. Following the Australian Research Council, Midmore (2017) defined research impact as:

The demonstrable contribution that research makes to the economy, society, culture, national security, public policy or services, health, the environment, or quality of life, beyond contributions to academia.

Agro-Food Research Organizations (‘AFRO’ thereafter) place increasing emphasis on implementing monitoring and evaluation (M&E) tools to assess the socio-economic impacts of R&D efforts, which are mainly driven by the four *‘As’* of advocacy, accountability, allocation, and analysis (Morgan et al. 2017). Nevertheless, AFRO are facing challenging issues to develop research impact assessment (RIA) mainly due to data scarcity, time lags between R&D outputs and impact, complexity of the innovation process, various actors involved in R&D activities, time-consuming evaluation techniques, and the need for flexible, robust and standardized metrics. On the other hand, RIA methodological improvements are still needed to optimize data collection procedures, to refine existing impact measures and to derive reliable and comprehensive lessons (Guesmi and Gil 2018).

Our discussion with different experts on RIA and actors from AFRO as well as published research studies that provide empirical evidence of institutional practices in the evaluation of research outputs and impacts, highlight a wide consensus on the relevance of RIA in terms of M&E performance of the AFRO and learning purposes. In contrast to the health research field, the culture of RIA is still at its early stage in AFRO. Commitment, Coordination and Collaboration are three prerequisite “C” keywords to integrate RIA culture within science organizations.

The authors would like to emphasize that making the RIA culture more common for both public and private actors requires designing a system of indicators that allows measuring the impact of change, technology and innovation not only in the sector, but in society. Impact measures should account for the multidimensional aspects of the agro-food sector (economic, territorial, health, social, political, environmental, capacity building) going beyond the three traditional pillars of sustainability (economic, social, environmental). The incorporation of RIA process into science organizations would provide a better evaluation platform to make science more effective for agro-food sector, to increase and to understand the impact of R&D. Putting into practice RIA activities is important to academic researchers, professionals, policy-makers and society to underpin a robust information sharing system between key actors. For academic actors, RIA may improve and demonstrate the benefits of their scientific outputs through getting regular and valuable feedback from key users of the research results. The business industry and society looked for the socio-economic value of science which could be obtained from new knowledge and technology transfer. Finally, for stakeholders and R&D funders it is important since accountability for and demonstration of impact gained from their investments has been substantially increased.

Alternative methodological frameworks have been used in the academic literature to evaluate R&D impacts ranging from qualitative and narrative analyses to sophisticated quantitative exercises. There is a growing consensus among the scientific community and practitioners that there is ‘no single impact’ to be evaluated and ‘no single best tool’ for doing so (Weißhuhn et al. 2017). Although the academic literature has been evolving and proposing new RIA techniques, there is still scope for further research to better articulate this concept. From this perspective, RIA would embrace mixed-method approaches at different levels of governance to enhance the evaluation of agro-food R&D impacts within research organizations. There is a need to develop coherent and inclusive methodological tools to assess the impact of both public and private R&D efforts toward sustainable development goals, this in itself would represent a step further towards improving the RIA because such framework does not currently exist, but is clearly needed.

To address these gaps, RIA should draw upon the participatory perspective as much as possible to produce socially robust knowledge (Nowotny et al. 2013) created in the context of practices and not only in the academic frame. This entails involving all key actors (e.g., funders, policy-makers, researchers, users, stakeholders) participating in R&D projects and actions including the early stages of the project (co-creation and co-design of research and analysis). Integrating all actors in the generation of R&D impact would help to decide what measures and indicators to implement, taking into account their needs, priorities, expectations and, on the other hand, to avoid ‘project fallacy’ problems and strengthen stakeholder relationships. This is key when developing a RIA strategy or a R&D impact portfolio for AFRO. This gives support to an evaluative culture of RIA across all actors and to have a global picture of R&D based on responsible research and innovation principles. By determining the ‘global’ R&D impact of AFRO, the latter can assess which R&D project or program could generate better societal benefits. In doing so, we can better identify supporting factors from successful innovation cases and overcome potential obstacles (scientific, technical and political) that could impede impact generation for future R&D projects or programs. In this way, RIA would be a strategic tool for learning purposes and management of R&D impact for AFRO.

Furthermore, AFRO have to define a RIA strategy (expected results, outputs and outcome) for their projects and research actions at the initial stage (ex-ante) in order to analyze the steps to be taken to achieve the desired impact and promote the vision of generating outstanding impacts related to research performance. Therefore, RIA will not be random, but also strive for research outcomes allowing to monitor R&D impact during the life cycle of the projects and to change the strategy if necessary.

The authors believe that AFRO should emphasize more effort to integrate a culture of systematization of good practices on impact generation, dissemination and assessment. A real cultural change in the AFRO is needed. AFRO should focus on generating valuable societal impacts that contribute to the four institutional ‘As’, not only focusing on research quality reflected in the excellence of scientific outputs.

AFRO are still facing limited capacity and lack of credibility of decision-makers to incorporate RIA culture in their impact evaluation agenda. While some of them are at advanced stages, others are at very early stages of integrating such culture. Overall, we would like to point out that RIA is a recent paradigm that has been receiving considerable attention in the agenda of the AFRO worldwide (e.g., USDA-USA, REF-United Kingdom, Embrapa-Brazil, INRAe-France, INIA-Uruguay, IRTA-Spain, Agresearch- New Zeeland, Teagasc-Ireland, among others). These organizations have demonstrated their ability to adapt to this new reality and blaze the path to establish a dynamic of periodical RIA within their evaluation system. In this way, an evaluation culture of R&D impacts can be evidenced by its applicability in other research fields and also its potential to be implemented in other AFRO in different countries. This could be achieved, in the spirit of collaboration between science organizations, through providing the opportunity to learn both across and within organizations and creating a network to improve the AFRO’s evaluation capacity, which in turn ensures sustainable future science impact.

Last and not least, RIA processes could become an evaluative practice within the AFRO to inform research policies and management about the societal impact of R&D results and to increase the impact of science. RIA encompasses both institutional learning capacity and external communication purposes. It is worth noting that the linkage between knowledge and innovation transfer and the societal impact of R&D is still overlooked. Therefore, further attention should be paid to how different knowledge and innovation transfer activities are contributing to disseminate R&D outcomes and to benefit a large number of target users. The next challenge is to develop RIA tools that strengthen the connection between ex-ante and ex-post impact monitoring and evaluation.
